# Market Segmentation by Motivations of Urban Forest Users and Differences in Perceived Effects

**DOI:** 10.3390/ijerph19010114

**Published:** 2021-12-23

**Authors:** Hyo-Jeong Byun, Byeong-Cheol Lee, Donghan Kim, Kwang-Hi Park

**Affiliations:** 1 Department of Liberal Arts College, Kyonggi University, Gyeonggi 15442, Korea; hjbyun@kgu.ac.kr; 2 Graduate School of Tourism, Event, and Convention Management, Kyonggi University, Seoul 03746, Korea; 2bclee@kgu.ac.kr; 3 Department of Convention and Hotel Management, College of Economics and Business Administration, Hannam University, Deajeon 34430, Korea; eastkorea@hnu.kr; 4 Department of Nursing, Gachon University, Incheon 21936, Korea

**Keywords:** urban forest, perceived effects, market segmentation, motivation

## Abstract

The purpose of this study aims at segmenting the urban forest users’ market by motivation and analyzing the difference in perceived effects of urban forests. Based on a literature review, the study selected seven motivating factors of urban forest users: experiential activity, relaxatin/healing, health management, escape from everday life, daily leisure, affinity toward nature. Data were collected online from 21 to 29 Sepember 2020 with urban forest visitors. We analyzed 878 questionnaires received from those with experience of visiting an urban forest within the previous 24 months. We performed a cluster analysis to classify the subjects according to the characteristics of urban forest utilization, and assigned them to four clusters (rest in nature, family leisure, passive participation, and multiple pursuit). An additional analysis was performed to determine intergroup differences, which revealed differences in perceived benefits and healing effects of urban forests as well as satisfaction. The results of this study provide implications for urban forest operation and strategy setup.

## 1. Introduction

Economic growth comes with increasingly serious exposure to various stressors and environmental pollutants in a complex social structure, and there is a growing desire for a healthy life [1,2]. In concert with improving standards of living and more time for leisure activities, people put more effort into closely linking recreation and leisure activities along with increased interest in health [3]. Additionally, there is a growing desire for the so-called wellbeing along, with increasing interest in mental health as well as physical health, such as mind–body relaxation and stability [4]. Driven by this shift in awareness, people’s desire for forest recreation has drastically increased in search of green spaces away from the artificial city environment to improve their quality of life, enjoying nature and relaxing [5].

Previous studies [1,6,7,8] have demonstrated that not only do forests provide opportunities for leisure activities, but they also have various educational and healing effects, improving both physical and psychological health. However, frequent and active use of rural woods and forests involves many constraints due to problems including accessibility. Therefore, availability of urban natural parks and neighborhood parks near residential areas is increasingly important. In particular, the research finding that the most frequently used type of forest experience is the village/urban forest walk (82.4%) [1] points to the high interest and utility rate of neighborhood green spaces.

Nature-based leisure activities have positive effects on emotional stability, stress relief, and depression and anxiety reduction [9,10,11,12,13], which eventually have the effect of enhancing the quality of life [14,15]. Modern-day people exposed to excessive work and life stress can improve their emotional stability through green spaces [16,17]. Activities in green spaces contribute not only to physical health, but also to stress relief, emotional stability, and improvement of social relationships [18,19,20]. In particular, with the spread of COVID-19, opportunities for outside activities, such as travel and exercise, have drastically decreased and the number of citizens suffering from so-called “corona blues” with complaints of depression due to stress and anxiety, has increased [21], On the other hand, with more and more citizens taking interest in forest resources, the number of citizens visiting nearby parks and woods has increased by 51% [22] and that of citizens visiting urban neighborhood national park accessible by car has increased by 42% [23].

Forests (and forest resources) have multiple effects and functions, and with people’s increasing awareness and understanding of the benefits and importance of urban forests in recent years, forest users’ needs are also changing [24]. In order to provide and improve the necessary experiences for various types of users, it is essential to better understand the motivations for visiting urban forests [25,26]. Most of the studies related to forest resources, such as urban forests, healing forests, national parks, and recreational forests, concern topics related to healing and health [27,28,29], and other studies concern environmental engineering approaches [30,31,32] and geographic standpoints such as urban development and regeneration [33,34]. Regarding the functions and benefits of urban forests as perceived by visitors, in-depth analysis has yet to be made and strategies to be setup to determine their effects on users according to the purpose of their visit. 

As mentioned above, the functions and importance of urban forests as leisure spaces in people’s lives are expanding, and users of urban forests are expected to visit urban forests for various purposes. This study was conducted to perform a market segmentation by the motivations for urban forest users and to establish effective management plans and strategies for urban forests by analyzing the differences in the benefits and effects of urban forests according to the segmented markets.

## 2. Theoretical Backgrounds

### 2.1. National and International Research Trends Related to (Urban) Forests

Although different criteria are applied for the scope of urban forests depending on the viewpoint, it includes vegetation planted in all spaces of large cities in the broader sense of the word, and refers to forests located in cities and adjacent areas in the narrower sense of the word [35]. Gray [36] classified urban forests into the types that can be managed directly and those that can be managed indirectly by local governments. The Korea Forest Service [37] classifies urban forests into living-area and non-living-area urban forests. In general, all green spaces with woods and vegetation (including street trees and park trees) in and around urban areas are considered urban forests, which comprehensively include all green spaces [38].

Studies on urban forests have been conducted at national and international levels from three perspectives: health and healing, environmental, and geographic purposes. First, to present some outcomes of the studies on urban forests related to health and healing [11,13,14,15,27,28,29,39,40], Kim [27] found that visitors prefer places where they can relax and change moods, such as meditation and contemplation and that users with diseases have keen interest in health promotion. Park and Koo [29] identified walking and hiking as well as recreation and relaxation as the main purposes of using urban forests and presented implications of urban forest utilization as forest healing spaces. A large number of studies (e.g., [11,13,14,15,28,40,41]) demonstrated healing effects of nature-based tourism activities on relieving depression, anxiety, and stress and regaining emotional stability, and emphasized the effects of nature-based activities on enhancing the quality of life. 

Second, studies conducted from an environmental perspective have investigated environmental effects of urban forests related to temperature and fine dust. Park and Lee [20] confirmed the effect urban green spaces on reducing the temperature in and around Seoul Forest. Kim [42] insisted on the need to expand urban forests as part of climate change response, and Jeong et al. [43] mentioned the importance of creating urban forests for fine dust reduction and derived a checklist for the items to be considered for urban forest creation. Baró et al. [32] emphasized that the ecosystem services, such as air purification, climate regulation, and air pollution reduction, provided by urban forests contribute to enhancing the quality of life in cities, and asserted the need for a wider green space.

Third, studies on urban forests conducted from a geographical perspective have examined geographic aspects related to urban development and urban regeneration. With regard to tourism development linking forests and tourism, Deng et al. [33] found that urban forests not only act as the main user attraction for visitors, but also play a role of complementing other tourist attractions in the city. Zhai et al. [34] suggested implications necessary for urban forest park planning and management by analyzing visitor characteristics such as accompanying type and staying type of urban forest users.

As examined above, urban forest-related studies have been conducted from various perspectives, but little research has been dedicated to in-depth multi-tiered investigation for efficient use of urban forests as citizens’ leisure spaces. This bias in research arose presumably due to the fact these studies investigated the aspects necessary in their respective academic disciplines, such as healthcare, geographic survey, and urban development, rather than the urban forests themselves. In other words, instead of discussing the functions of urban forests according to the motivations of urban forest users and their utilization plan, these studies conducted surveys to verify the effects of urban forests on people who visited urban forests for specific purposes such as healing or specific functions such as find dust and temperature reduction effects. With these approaches, it is difficult to properly understand various functions of urban forests and motivations of visitors. Likewise, with the results thus obtained, it would be difficult to set up strategies for effective urban forest utilization.

Against this background, this study aims to present the motivations and benefits for urban forest users from a broader standpoint, identify their characteristics, and propose implications for efficient management of urban forests from the perspective of urban forest users as well. 

### 2.2. Urban Forest Visit Motivations

Urban forest users visit urban forests for various reasons and purposes. Some seek places for contemplation, peace, and stillness, and others use urban forests for recreational activities [44]. Arnberger et al. [45] noted that stress reduction and meeting family and friends are also important motivations for visiting forests. With increase in the number of forest users, their demands are also changing as they become more aware of the benefits provided by urban forests and their importance [24], and it is essential to have a proper understanding of different motivations for visiting urban forests in order to provide experiences required by urban forest users and improve the service [25,26].

Previous studies conducted in Korea [31,46] identified the main purposes (motivations) for visiting urban forests as health maintenance, relaxation, affinity toward nature, escape from everyday life (city), and playing with children. In a study on eco-forest utilization behaviors [47], the purposes of relaxation/leisure and learning/education accounted for high proportions and family and friends/colleagues were found to be the most frequent accompanying persons. Kim and Lee [48] conducted a survey with people who participated in a forest experience program with their children for the purpose of improving the parent–child relationship, enhancing physical and mental health, and experiential learning and confirmed its beneficial effects in terms of emotional communication with children. Kwon and Lee [49] designed a questionnaire survey with 12 motivations for forest trail users and identified “clean air and health promotion” and “escape from urban life to enjoy leisure” as the most frequent motivations for forest visit. Drawing on these findings of previous studies, we applied items including relaxation/healing, affinity toward nature, daily leisure, health management, and various activities as urban forest visit motivations.

Unlike in the past, when the main function of forests and other green spaces was improving the comfort of the environment, the focus has shifted to providing citizens with opportunities to rest, improve their quality of life, and recharge their energy [50], and urban forests also provide various services and benefits [51,52,53,54]. In particular, with increased time for leisure, urban residents have a strong desire to pursue a healthy leisure life, and a significant portion of their leisure demand is associated with forests [55], and there is a growing demand for urban forests which are main natural resources in urban areas [56]. In other words, urban forests are recognized for their value because they can enjoy various benefits of forests within the living sphere, and they can achieve the effects of landscape and environmental improvement at the urban [57].

### 2.3. Benefits of Urban Forest

Since urban forests provide urban residents with a range of benefits and the urban areas with attractive sceneries and improved environment, their values are well-appreciated [57]. At the individual level, they urban forests provides spaces for relaxation and leisure activity and opportunities for emotional stability and health promotion, which are highly appreciated by visitors [3,58,59,60,61]. Furthermore, urban forests’ healing and health-promoting effects, which are slightly inferior to natural forests, have been demonstrated by many studies [27,33,62,63].

Shin et al. [8] reported a significant positive effect of forest experience on mental health, and in an experimental study with college students, Park [7] verified an invigorating effect of forest scenery and hiking on anxiety, tension, depression, and fatigue by mitigating related emotions. In particular, forests are effective in relieving chronic stress [64], and repeated experiences of convalescence in nature have a cumulative effect, such that the positive effect of forest experience in everyday life increases proportionally to such experiences [65]. Emotional health fortified through nature-based activities has been verified to improve quality of life [14,15,66].

Environmental effects of urban forests and other green spaces, such as heat island mitigation and fin dust reduction, have also been demonstrated by many studies [30,32], resulting in health promotion and thus enhancing life satisfaction of urban residents health outcomes. Drawing on these research findings, we comprehensively considered not only the physical, environmental, and educational aspects, but also the healing effects in the discussion of the functions and benefits of urban forests.

## 3. Methodology

### 3.1. Research Subject and Data Collection

The subject of this study were urban forest users, including both park-type and industry–academia-type forests. The survey for this study was conducted from 21 to 29 September 2020 with Seoul and Incheon citizens with experience of visiting urban forest within the past 24 months. For data collection, a professional research agency (Korea Data Research Center) conducted an online questionnaire survey, in which a total of 1000 questionnaires were distributed to the individuals selected through non-probability/quota sampling. After excluding 122 questionnaires from people without experience of visiting urban forests, a total of 878 questionnaires were used for data analysis. The questionnaire consisted of a 7-point Likert scale.

### 3.2. Measurement Items and Questionnaire Structure

Table 1 outlines the measurement items and questionnaire structure setup based on previous studies.

### 3.3. Research Hypotheses

This study was conducted to draw implications for revitalizing the urban forest utilization by deriving efficient utilization and management modalities based on the analysis results of the differences in perceived effects among urban forest users under the assumption that urban forest users’ perceived effects of urban forests would vary according to their visit motivation. Accordingly, the following research hypotheses were formulated:

**Hypothesis** **1.** *The perceived benefits of urban forests will vary according to the motivations of urban forest visit.*

**Hypothesis** **2.** *The perceived healing effects of urban forests will vary according to the motivations of urban forest visit.*

**Hypothesis** **3.** *The satisfaction and revisit intention will vary according to the motivations of urban forest visit.*

## 4. Result of analysis

### 4.1. Analysis of Demographic Characteristics 

In this study, a total of 878 questionnaires collected from urban residents with experience of visiting urban forests within the past 24 months were used for analysis. Frequency analysis was performed to identify the subjects’ demographic characteristics, which resulted as follows: in gender distribution, women accounted for 50.9% (n = 447) and men 49.1% (n = 431); in marital status, married accounted for 59.7% (n = 524), single 38.5 % (n = 338) and other 1.8% (n=16); the most frequent age group was 50–59 (20.6%), followed 40–49 (19.7%), 20–29 (18.7%) and 30–39 (18.6%); in terms of education level, college graduates accounted for the highest proportion (59.1%), followed by high school graduates (24.3%) and postgraduate or higher (12.8%); the most frequent occupation was office/technical jobs (39.5%), followed by housewives (15.8%), students (13.6%), and professionals (10.3%) (Table 2).

### 4.2. Urban Forest Utilization Behavior

A great majority, 71.2% (n = 625), of the respondents visited urban forests on weekends (Saturday–Sunday), followed by on weekdays (Mon–Thursday) with 23.6% (n = 207), with the remaining 3.6% (n = 32) found to visit urban forests every day. The most frequent time of the day for visiting urban forests was in the afternoon (14:00 to 18:00) with 358 (40.8%), followed by 231 (26.3%) in before noon (09:00–12:00) and 117 (13.3%) in the evening (18:00 to 21:00). In terms of accompanying persons, 451 respondents (51.4%) answered that they visit forests with family/relatives, followed by friends/lovers (218, 24.8%), and alone (172, 19.6%) who visit alone. As for information acquisition route, the largest number of respondents (541, 25.0%) answered that they knew about urban forests from past experience, followed by recommendations (430, 19.9%), online blogs/cafes (409, 18.9%), and social media (250, 11.6%) in that order (Table 3).

### 4.3. Reliability and Validity Analysis of Measurement Items

Exploratory factor analysis and reliability analysis were performed to analyze the reliability and validity of the measurement items used in this study. For factor analysis, varimax rotation was performed as a principal component analysis, and the number of factors was analyzed based on an eigenvalue of 1.0. Analysis for reliability testing was performed using Cronbach’s α coefficients.

A 7-point Likert scale was used for rating each measurement item of the questionnaire. As a result of factor analysis, a total of 7 factors were extracted as the urban forest visit motivations. The overall KMO value of the extracted factors was found to be excellent with 0.909, and the validity of factor analysis was significant at *p* < 0.001. Convergent validity was established, with the factor loadings of all items exceeding 0.5, and the internal reliability of each factor was also verified, with the Cronbach’s α value arranging from 0.791 to 0.894 (Table 4).

Factor analysis of urban forest benefits yielded three factors. The KMO value of the extracted factors was very high with 0.893, and the validity of factor analysis was significant at *p* < 0.001. Convergent validity was established, with all factor loading values exceeding 0.5, and the internal reliability of each factor was also verified, with the Cronbach’s α values ranging from 0.780 to 0.862 (Table 5).

Factor analysis of healing effect of urban forests yielded one factor. The KMO value of the extracted factor was very high with 0.919, and the validity of factor analysis was significant at *p* < 0.001. Convergent validity was established, with all factor loading values exceeding 0.5, and the internal reliability of the factor was also verified (Cronbach’s α = 0.928) (Table 6).

Factor analysis of satisfaction yielded two factors (Table 7). The KMO value of the extracted factors was very high with 0.850, and the validity of factor analysis was significant at *p* < 0.001. Convergent validity was established, with all factor loading values exceeding 0.5, and the internal reliability of each factor was also verified, with the Cronbach’s α values calculated at 0.878 and 0.788, respectively. 

Factor analysis of revisit intention yielded one factor. The KMO value of the extracted factor was calculated at 0.705, and the validity of factor analysis was significant at *p* < 0.001. Convergent validity was established, with all factor loading values exceeding 0.5, and the internal reliability of the factor was also verified (Cronbach’s α = 0.814) (Table 8).

### 4.4. Clustering According to the Urban Forest Visit Motivations

In order to cluster the subjects according to the visit-related characteristics based on the seven domains extracted by factor analysis of urban forest visit motivations, namely, experiential activity, relaxation/healing, health management, escape from everyday life, daily leisure, family activities, and affinity toward nature. The optimal number of clusters was set at four as a result of hierarchical clustering performed on the 878 valid samples, followed by the procedure of quick clustering K-means to identify four clusters.

As a result of one-way ANOVA and post hoc test, which were performed to determine the names of the four clusters derived, significant differences were found in all factors at the significance level of <0.001 (Table 9).

Taking into account the characteristics of each cluster, the four clusters were named: rest in nature (Cluster 1 (C-a)), family leisure, (Cluster 2 (C-b)) passive participation (Cluster 3 (C-c)), and multiple pursuit (Cluster 4 (C-d)). As a result of cluster analysis, relaxation/healing showed high mean scores across the clusters, which is thus interpreted as the motivation basically pursued by urban forest users. Characteristics of each cluster are as follows: C-a has a high mean score for affinity toward nature and attaches importance to healing using natural resources as a major urban forest visit motivation; C-b has a high mean score for family activity and is considered a group using urban forests for activities such as family outing or camping; C-c has lower mean scores across the items, showing passive attitudes toward urban forest visit motivations; and C-d has higher mean scores across the items as a group driven by multiple visit motivations.

### 4.5. Research Hypotheses Testing

- Hypothesis 1

One-way ANOVA was performed to test significant intergroup differences in the perceived benefits of urban forests according to visit motivations, and the analysis results are outlined in Table 10.

Regarding the physical benefits of urban forests, significant differences were observed between C-a and C-b/C-d (i.e., C-a and C-b as well as C-a and C-d), whereby C-a’s perception of physical benefits was the highest, followed by C-d and C-b. Significant differences were also observed between C-b and C-c. Intergroup differences were also confirmed between C-c and C-d.

Regarding the environmental benefits of urban forests, intergroup differences were observed among C-a, C-b, and C-d, whereby C-a’s perception of environmental benefits was the highest. Statistically significant differences were observed between C-c/C-d and C-a, with C-c and C-d verified to perceive the environmental benefits more acutely than C-b. Lastly, regarding the educational benefits of urban forests, intergroup differences were observed between C-a and C-b/C-d, as well as C-a and C-c and C-c and C-d.

- Hypothesis 2

One-way ANOVA was performed to test significant intergroup differences in the perceived healing effects of urban forests according to visit motivations, and the analysis results are outlined in Table 11.

As a result, intergroup differences were observed with F-value of 47.457(*p* = 0.000). More specifically, differences were observed between C-a and C-b/C-d, and statistically significant differences were observed between C-c and C-b/C-d.

- Hypothesis 3

One-way ANOVA was performed to test significant intergroup differences in the perceived satisfaction with urban forests according to visit motivations, and the analysis results are outlined in Table 12.

As a result, statistically significant (*p* < 0.000) intergroup differences were observed in environmental satisfaction (F = 24.280) and facility satisfaction (F = 47.519). Intergroup differences were also observed in revisit intention (F = 30.894; p=0.000). Specifically, differences in environmental satisfaction existed between C-a and C-b/C-d, between C-b and C-c, and between C-c and C-d. As for facility satisfaction, C-a showed differences from all other clusters, as did C-b.

As regards revisit intention, C-a showed differences from C-b and C-d, and C-d from all other clusters. 

## 5. Conclusions

### 5.1. Summary of the Study Results

The primary aim of this study was to derive efficient urban forest utilization measures and strategies through market segmentation by identifying urban forest users’ visit motivations and perceived benefits against the background of increasing importance of forest resources in and around urban areas. The results of the study can be summarized as follows.

First, the cluster analysis, which was performed to segment the market according to the urban forest visit motivations, yielded four clusters: rest in nature (C-a), family leisure (C-b), passive participation (C-c), and multiple pursuit (C-d). 

Second, intergroup differences in the perceived benefits of urban forests were analyzed, which resulted in the finding that intergroup differences were present in all three beneficial aspects, namely, physical, environmental, and educational benefits. As regards physical benefits, C-a showed the highest mean score (5.86) in perceived physical benefit, followed by C-c, C-d, and C-b. C-a also showed the highest mean score (5.49) in perceived environmental benefits, and C-b the lowest mean score. Compared with physical and environmental benefits, the scores for educational benefits were lower across the clusters, especially in C-b (4.01). These results suggest that C-a is highly aware of physical and environmental benefits because it is the cluster that has a particularly strong motivation for relaxation/recreation using natural resources, coupled with a high motivation to escape from polluted everyday life, and attaches great value to health management. In contrast, C-d scored higher than all other clusters in perceived educational benefits (4.87), presumably because their attach higher value to culture and art spaces or forest-based education in urban forests than to the benefits from natural resources.

Third, intergroup differences in the perceived healing effects of urban forests were analyzed. As a result, intergroup differences were observed, whereby C-a was found to have the highest perceived healing effects (5.99), followed by C-d, C-b, and C-d. This result suggests that C-a perceived the healing effects of urban forests more strongly than other clusters because it has high motivations for relaxation/healing, health management, and affinity toward nature. What is worth noting is that C-c showed a high level of perceived healing effects of urban forests (5.82) despite its overall low scores in urban forest visit motivations, presumably because of the stress-relieving and health-promoting effects of urban forests.

Fourth, an analysis of intergroup differences in satisfaction verified statistically significant intergroup differences in both environmental satisfaction and facility satisfaction. C-c (5.91) scored highest in environmental satisfaction, followed by C-a (5.83), C-d (5.54), and C-b (5.32) Environmental satisfaction is high across the clusters. C-c, in particular, which had low scores across the urban forest visit motivations, seems to be satisfied with the fact alone that they can enjoy a safe and pleasant walk in nearby urban forests. However, it showed a lower level of satisfaction in facility satisfaction compared with environmental satisfaction. In contrast, C-d (4.35) showed the highest level of facility satisfaction, presumably because, as a cluster driven by high urban forest visit motivations, they have actively experienced various facilities of urban forests.

Fifth, statistically significant differences were also observed in revisit intention, whereby C-a (5.83) showed the highest mean score, and C-b (5.07) the lowest. C-a’s highest revisit intention score seems to be associated with its overall high mean scores. As regards the high mean scores obtained by C-a (5.83) and C-c (5.71) in revisit intention, that of C-a may be explained by the easy accessibility of urban forests to enjoy relaxation and healing using forest resources, and that of C-c may be explained by the perception of various benefits provided by urban forests despite its low visit motivations.

### 5.2. Implications

This study aimed to contribute to setting up effective urban forest utilization measures and strategies through market segmentation. Taking the results of this study together, we propose the following theoretical and practical implications.

First, it is necessary to provide various experiences and events in the management of urban forests. Among the benefits of urban forest, citizens’ perceptions of of physical and environmental benefits were relatively high, while those of educational benefits were comparatively lower. This reflects the fact that citizens are familiar with the environmental and physical benefits provided by urban forests as typical functions of forests. While the educational benefits, traditionally less known as benefits provided by urban forests, are less perceived by all other clusters, C-c, which showed the lowest urban forest visit motivations, scored highest in perceiving the educational benefits of urban forest. This generally low perception of the educational benefits of urban forests highlights the need to actively promote various experiences and events provided by urban forests as opposed to their past image as parks. At the same time, such benefits can act as an element to fortify urban residents desire to visit forests in their vicinity. Therefore, it seems to be necessary to provide various events so that more citizens can actively use urban forests. It can also be expected to expand the role of urban forests by involving or utilizing local residents in the planning and designing such events and using them as onsite locations for forestry education. 

Second, it is necessary to develop programs conducive to enhancing the healing effects for visitors. All groups showed high perceptions of the healing effects of urban forests, which is one of the major motivations for visiting urban forests. In particular, from the fact that even C-c, the passive participation cluster with generally low urban forest visit motivations, showed a high level of perceived healing effects of the urban forests highlights the need to develop forest healing programs as one of the most important functions of urban forest. In order to enlarge the urban forest user base and enhance user satisfaction, it is necessary to develop not only expert-designed, but also visitor-designed programs.

Third, it is necessary to install a variety of facilities in urban forests and set up utilization strategies. According to the results of this study, the most important motivations for visiting urban forests are relaxation/healing and affinity toward nature. For city dwellers, who mostly live in a space surrounded by forests of high-rise apartment buildings, urban forests are valuable assets just for the fact that they allow them to experience nature in their vicinity and feel relaxation and healing in nature. However, various other motivations were also verified in this study, and in order to satisfy urban forest users with many different motives, it is necessary to provide them with a wide range of physical facilities catering for their needs. Since the intensity of activity in urban forests can have a greater effect on life satisfaction than the duration of stay there [67,68], it is crucial to create a physical environment where visitors feel like staying longer and enjoying more. In other words, it is of vital importance to install age-specific facilities (for children and seniors) and spaces accessible to people with physical disabilities. Urban forest users’ satisfaction can be enhanced by providing them with various facilities on abundant green space, such as appropriate exercise facilities, relaxation and convenience facilities, and facilities that can be used with pets.

Lastly, it is necessary to launch web-based active publicity and marketing campaigns. The most frequent information acquisition route of urban forest users was their past experience, and 11.6% obtained information through social media, which is exceptionally low, given the recent increase in the use of social media. The low rate of information acquisition through social media can also be interpreted as a low interest in urban forests among young people. Therefore, more active social media-based publicity and marketing activities are required to induce more young people to take interest in activities and events in urban forests.

Urban forests are gaining traction as neighborhood leisure spaces of urban residents, going beyond the past image and function of neighborhood parks. They have become not only spaces for healing and health management using natural resources, but also as cultural arena where all types of citizens enjoy leisure, from families to lovers. To this end, basic facility management for safety (security) and environmental hygiene should be first ensured, along with the various approaches presented above, and consistent and effective management must also be ensured under the concerted efforts of local governments and residents.

Despite several important implications for urban forest utilization measures and strategies drawn in this study, two aspects may be pointed out as limitations of this study. First, the sample of this study is regionally limited to Seoul and Incheon. Follow-up research will have to expand its target population to other regions. Second, this study did not reveal a causal relationship regarding user satisfaction. Therefore, it is necessary to conduct a follow-up study with a focus on revealing the causal relationship between user motivations and user satisfaction with the benefits offered by urban forests. Finally, according to a previous study [69] stating that there are differences in air quality perception according to age groups, it is thought that a research design that considers age groups would be more meaningful.

## Figures and Tables

**Table 1:** Measurement items and questionnaire design.

Category	Measurement Item	No. of Items	References
Type of urban forest users	Visit motivation	30	[29,31,33,44,45,46,55,67,68]
	Accompany, frequency of use, preference, duration, information route, satisfaction, revisit intention	19	
Functions of urban forests	Benefits of urban forests	11	[27,39,41,69]
	Healing effects	9	
Demographic characteristics	Gender, age, place of residence, education, marital status, child status, mean monthly household income, occupation	9	-

**Table 2:** Demographic characteristics.

Category	Frequency (%)	Category	Frequency (%)
**Gender**		**Marital status**	
Male	431(49.1)	Married	524(59.7)
Female	447(50.9)	Unmarried	338(38.5)
**Age**		Others	16(1.8)
10–19	49(5.6)	**Education level**	
20–29	164(18.7)	<High school	34(3.9)
30–39	163(18.6)	High school	213(24.3)
40–49	173(19.7)	University	519(59.1)
50–59	181(20.6)	≥Postgraduate	112(12.8)
≥60	148(16.9)	**Occupation**	
**Monthly household income**		Office/Technical	347(39.5)
<100	27(3.1)	Professional	90(10.3)
<200	55(6.3)	Self-owned	43(4.9)
<300	114(13.0)	Sales/Service	56(6.4)
<400	128(14.6)	Freelancer	14(1.6)
<500	126(14.4)	Housewife	139(15.8)
<600	145(16.5)	Student	119(13.6)
≥601	283(32.1)	Unemployed	51(5.8)
		Others	19(2.2)
Total	878(100.0)	Total	878(100.0)

**Table 3:** Urban forest utilization analysis results.

Category	Frequency (%)	Category	Frequency (%)
**Day of the week**		**Accompany**	
Weekday (Monday–Thurday)	207(23.6)	Alone	172(19.6)
Friday	14(1.6)	Family/relatives	451(51.4)
Weekend (Saturday–Sunday)	625(71.2)	Friend/lover	218(24.8)
Everyday	32(3.6)	Colleague/club	34(3.9)
**Time of the day**		Companion dog	3(0.3)
Dawn (05–07)	11(1.3)	**Information route**	
Early morning (07–09)	48(5.5)	Blog/cafe	409(18.9)
Before noon (09–12)	231(26.3)	Internet news	175(8.1)
Midday (12–14)	104(11.8)	Recommendation	430(19.9)
Afternoon (14–18)	358(40.8)	Broadcaster (TV, radio)	189(8.7)
Evening (18–21)	117(13.3)	Tourist guide/magazine	94(4.3)
Night (after 21)	9(1.0)	Social media	250(11.6)
		Past experience	541(25.0)
		None	75(3.5)
Total	878(100.0)	Total	878(100.0)

**Table 4:** Reliability and validity test results for urban forest visit motivations (n = 878).

Factor	Measurement Item	Factor Loading	Eigenvalue (Cumulative)%	Cronbach’s α
Experiential activity	Performance watching Exhibitions (expositions) Participation in events Forest-related activities Landscape photography	0.847 0.834 0.830 0.669 0.449	13.057 (13.057)	0.894
Relaxation /Healing	Escape from everyday life Stress relief Recharge Time with nature	0.759 0.739 0.720 0.631	11.245 (24.302)	0.878
Health management	Health maintenance Light exercise Disease prevention Healing illness Health/healing programs Walking and hiking	0.810 0.787 0.745 0.609 0.501 0.485	10.598 (34.901)	0.826
Escape from everyday life	Escape from fine dust Fresh air intake Escape from noise Feeling the cool temperature	0.764 0.738 0.7380.615	10.063 (44.964)	0.816
Daily leisure	Date Dog walking Walking along the bike path Use of amenities Use of nearby cafes/restaurants	0.771 0.720 0.720 0.599 0.590	9.478 (54.441)	0.845
Affinity toward nature	Nature and green space use Landscape appreciation Communication with nature	0.765 0.745 0.570	7.179 (61.620)	0.791
Family activity	Family outing Family camping	0.868 0.796	5.580 (67.200)	0.794

KMO = 0.909, χ² = 14707.158, df = 435 (*p* < 0.000).

**Table 5:** Reliability and validity test results for urban forest benefits (n = 878).

Factor	Measurement Item	Factor Loading	Eigenvalue (Cumulative)%	Cronbach’s α
Physical benefits	Landscape Relaxation and leisure Nature and green space Exercise space Healing and health promotion	0.795 0.787 0.766 0.635 0.521	25.715 (25.715)	0.839
Environmental benefits	Air purification Heat (heat island) mitigation Noise mitigation Environment protection	0.808 0.768 0.750 0.680	25.600 (51.314)	0.862
Educational benefits	Culture and art space Forest education	0.832 0.805	20.495 (71.810)	0.780

KMO = 0.893, χ² = 5882.786, df= 55 (*p* < 0.000).

**Table 6:** Reliability and validity test results for healing effect (n = 878).

Factor	Measurement Item	Factor Loading	Eigenvalue (Cumulative)%	Cronbach’s α
Healing effect	Vitality maintenance Health management Mental and physical stability Stress relief Exercise effect enhancement Recharging Change of moods Immunity improvement Disease prevention	0.858 0.841 0.824 0.818 0.818 0.815 0.806 0.740 0.702	64.629 (64.629)	0.928

KMO = 0.919, χ² = 6588.240, df= 36 (*p* < 0.000).

**Table 7:** Reliability and validity test results for satisfaction (n = 878).

Factor	Measurement Item	Factor Loading	Eigenvalue (Cumulative)%	Cronbach’s α
Environmental Satisfaction	Safety Comfort Hiking trail Convenience Accessibility	0.835 0.794 0.760 0.668 0.664	4.367 (33.887)	0.878
Facility satisfaction	Experience (education) program Healing program Facilities for pets Sports facilities	0.889 0.879 0.709 0.579	1.395 (64.015)	0.788

KMO = 0.850, χ² = 3332.913, df = 36 (*p* < 0.000).

**Table 8:** Reliability and validity test results for revisit intention (n = 878).

Factor	Measurement Item	Factor Loading	Eigenvalue (Cumulative)%	Cronbach’s α
Revisit intention	Intention to visit more often Intention to visit other urban forests Recommendation intention	0.873 0.872 0.818	2.193 (73.095)	0.814

KMO = 0.705, χ² = 929.317, df = 3 (*p* < 0.000).

**Table 9:** Cluster analysis according to the urban forest visit motivation.

Factor	Cluster 1 (n = 236)	Cluster 2 (n = 266)	Cluster 3 (n = 179)	Cluster 4 (n = 197)	F	*p*
	Mean	Mean	Mean	Mean		
Experiential activity	3.26	3.81	2.35	5.06	324.508	0.000
Relaxation/ Healing	5.92	5.32	4.76	6.03	133.889	0.000
Health management	5.22	4.40	3.97	5.61	176.409	0.000
Escape from everyday life	5.37	4.34	3.58	5.74	280.608	0.000
Daily leisure	2.31	3.27	2.25	4.42	233.101	0.000
Family activity	3.56	4.95	2.45	5.47	325.921	0.000
Affinity toward nature	5.61	5.03	4.03	5.91	221.992	0.000
Cluster name	RN	FL	PP	MP	-	-

RN: rest in nature, FL: family leisure, PP: passive participation, MP: multiple pursuit.

**Table 10:** Mean difference (one-way ANOVA) test for intergroup differences in the benefits.

Category	Group (I)	Group (J)	Mean Dif. (I–J)	Standard Error	*p*-Value	F-Value
Physical benefits	RN (a) m = 5.86	b	0.46160	0.076	0.000 ***	19.138 (*p* = 0.000)
		c	0.15192	0.066	0.154	
		d	0.43709	0.071	0.000 ***	
	FL (b) m = 5.40	a	−0.46160	0.076	0.000 ***	
		c	−0.30969	0.071	0.000 ***	
		d	−0.02452	0.076	0.991	
	PP (c) m = 5.71	a	−0.15192	0.066	0.154	
		b	0.30969	0.071	0.000 ***	
		d	0.28517	0.066	0.000 ***	
	MP (d) m = 5.42	a	−0.43709	0.071	0.000 ***	
		b	0.02452	0.076	0.991	
		c	−0.28517	0.066	0.000 ***	
* scheffe: a≠b, d/b≠c/c≠d						
Environmental benefits	RN (a) m = 5.49	b	.69968	0.098	0.000 ***	25.535 (*p* = 0.000)
		c	0.01969	0.086	0.997	
		d	0.41782	0.092	0.000 ***	
	FL (b) m = 4.79	a	−0.69968	0.098	0.000 ***	
		c	−0.67999	0.091	0.000 ***	
		d	−0.28186	0.098	0.041 *	
	PP (c) m = 5.47	a	−0.01969	0.086	0.997	
		b	0.67999	0.091	0.000 ***	
		d	0.39813	0.085	0.000 ***	
	MP (d) m = 5.07	a	−0.41782	0.092	0.000 ***	
		b	0.28186	0.098	0.041 *	
		c	−0.39813	0.085	0.000 ***	
* scheffe: a≠b, d/b≠c≠d						
Educational benefits	RN (a) m = 4.75	b	0.74049	0.109	0.000 ***	34.980 (*p* = 0.000)
		c	−0.11910	0.095	0.664	
		d	0.57036	0.102	0.000 ***	
	FL (b) m = 4.01	a	−0.74049	0.109	0.000 ***	
		c	−0.85959	0.101	0.000 ***	
		d	−0.17013	0.108	0.482	
	PP (c) m = 4.87	a	0.11910	0.095	0.664	
		b	0.85959	0.101	0.000 ***	
		d	0.68946	0.094	0.000 ***	
	MP (d) m = 4.18	a	−0.57036	0.102	0.000 ***	
		b	0.17013	0.108	0.482	
		c	−0.68946	0.094	0.000 ***	
* scheffe: a≠b, d/b≠c/c≠d						

RN: rest in nature, FL: family leisure, PP: passive participation, MP: multiple pursuit. * *p* < 0.05, *** *p* < 0.001.

**Table 11:** Mean difference (one-way ANOVA) test for intergroup differences in healing effects.

Category	Group (I)	Group (J)	Mean Dif. (I–J)	Standard Error	*p*-Value	F-Value
Healing effect	Rest in nature (a) m = 5.99	b	0.75947	0.075	0.000 ***	47.457 (*p* = 0.000)
		c	0.16419	0.065	0.099	
		d	0.57605	0.071	0.000 ***	
	Family leisure (b) m = 5.23	a	−0.75947	0.075	0.000 ***	
		c	−0.59528	0.070	0.000 ***	
		d	−0.18341	0.075	0.112	
	Passive participation (c) m = 5.82	a	−0.16419	0.065	0.099	
		b	0.59528	0.070	0.000 ***	
		d	0.41187	0.065	0.000 ***	
	Multiple pursuit (d) m = 5.65	a	−0.57605	0.071	0.000 ***	
		b	0.18341	0.075	0.112	
		c	−0.41187	0.065	0.000 ***	
* scheffe: a≠b, d/c≠b, d						

* *p* < 0.05, *** *p* < 0.001.

**Table 12:** Mean difference (one-way ANOVA) test for intergroup differences in satisfaction and revisit intention.

Category	Group (I)	Group (J)	Mean Dif. (I–J)	Standard Error	*p*-Value	F-Value
Environmental satisfaction	Rest in nature (a) m = 5.83	b	0.50567	0.082	0.000 ***	24.280 (*p* = 0.000)
		c	−0.08183	0.072	0.731	
		d	0.29382	0.078	0.003 **	
	Family leisure (b) m = 5.32	a	−0.50567	0.082	0.000 ***	
		c	−0.58750	0.077	0.000 ***	
		d	−0.21186	0.082	0.085	
	Passive participation (c) m = 5.91	a	0.08183	0.072	0.731	
		b	0.58750	0.077	0.000 ***	
		d	0.37565	0.072	0.000 ***	
	Multiple pursuit (d) m = 5.54	a	−0.29382	0.078	0.003 **	
		b	0.21186	0.082	0.085	
		c	−0.37565	0.072	0.000 ***	
* scheffe: a≠b, d/b≠c/c≠d						
Facility satisfaction	Rest in nature (a) m = 4.30	b	0.42471	0.107	0.001 ***	47.519 (*p* = 0.000)
		c	−0.56775	0.092	0.000 ***	
		d	0.31999	0.101	0.019 *	
	Family leisure (b) m = 3.88	a	−0.42471	0.107	0.001 ***	
		c	−0.99246	0.098	0.000 ***	
		d	−0.10472	0.107	0.811	
	Passive participation (c) m = 3.98	a	0.56775	0.092	0.000 ***	
		b	0.99246	0.098	0.000 ***	
		d	0.88774	0.092	0.000 ***	
	Multiple pursuit (d) m = 4.35	a	−0.31999	0.101	0.019 *	
		b	0.10472	0.107	0.811	
		c	−0.88774	0.092	0.000 ***	
* scheffe: a≠b, c, d/c≠a, b, d						
Revisit intention	Rest in nature(a) m = 5.83	b	0.76029	0.089	0.000 ***	30.666 (*p* = 0.000)
		c	0.11645	0.077	0.521	
		d	0.42620	0.084	0.000 ***	
	Family leisure (b) m = 5.07	a	−0.76029	0.089	0.000 ***	
		c	−0.64383	0.083	0.000 ***	
		d	−0.33408	0.089	0.003 **	
	Passive participation (c) m = 5.71	a	−0.11645	0.077	0.521	
		b	0.64383	0.083	0.000 ***	
		d	0.30975	0.077	0.001 ***	
	Multiple pursuit(d) m = 5.50	a	−0.42620	0.084	0.000 ***	
		b	0.33408	0.089	0.003 **	
		c	−0.30975	0.077	0.001 ***	
* scheffe: a≠b, d/b≠c≠d						

* *p* < 0.05, ** *p* < 0.01, *** *p* < 0.001.

## Data Availability

Not applicable.
